# Mitochondria in hypoxic pulmonary hypertension, roles and the potential targets

**DOI:** 10.3389/fphys.2023.1239643

**Published:** 2023-08-14

**Authors:** Yumei Geng, Yu Hu, Fang Zhang, Yajun Tuo, Rili Ge, Zhenzhong Bai

**Affiliations:** ^1^ Key Laboratory of High Altitude Medicine (Ministry of Education), Key Laboratory of Application and Foundation for High Altitude Medicine Research in Qinghai Province (Qinghai-Utah Joint Research Key Lab for High Altitude Medicine), Research Center for High Altitude Medicine, Qinghai University, Xining, China; ^2^ Department of Respiratory and Critical Care Medicine, Qinghai Provincial People’s Hospital, Xining, China; ^3^ Department of Pharmacy, Qinghai Provincial Traffic Hospital, Xining, China

**Keywords:** hypoxia, mitochondria, hypoxic pulmonary hypertension, mitochondrial dynamics, mitophagy, mtROS, target mitochondria

## Abstract

Mitochondria are the centrol hub for cellular energy metabolisms. They regulate fuel metabolism by oxygen levels, participate in physiological signaling pathways, and act as oxygen sensors. Once oxygen deprived, the fuel utilizations can be switched from mitochondrial oxidative phosphorylation to glycolysis for ATP production. Notably, mitochondria can also adapt to hypoxia by making various functional and phenotypes changes to meet the demanding of oxygen levels. Hypoxic pulmonary hypertension is a life-threatening disease, but its exact pathgenesis mechanism is still unclear and there is no effective treatment available until now. Ample of evidence indicated that mitochondria play key factor in the development of hypoxic pulmonary hypertension. By hypoxia-inducible factors, multiple cells sense and transmit hypoxia signals, which then control the expression of various metabolic genes. This activation of hypoxia-inducible factors considered associations with crosstalk between hypoxia and altered mitochondrial metabolism, which plays an important role in the development of hypoxic pulmonary hypertension. Here, we review the molecular mechanisms of how hypoxia affects mitochondrial function, including mitochondrial biosynthesis, reactive oxygen homeostasis, and mitochondrial dynamics, to explore the potential of improving mitochondrial function as a strategy for treating hypoxic pulmonary hypertension.

## 1 Introduction

Oxygen is crucial to generates ATP through by accepting electrons from a proton gradient in mitochondria. In the sea level, oxygen is ample for fuel metabolism, but in hypoxia, the oxygen is deprived and which can be caused by high altitudes, chronic obstructive pulmonary disease (COPD), anemia, carcinoma, and cardiovascular diseases (CVDs). Moreover, reprogrammed mitochondrial activities, by enhancing glycolysis to produce non-aerobic ATP production, is a common strategy to adaption to hypoxia in the multiple cells, in which hypoxia-inducible factors (HIFs) play a key role to maintain oxygen homeostasis. The cellular oxygen sensing system in cases of chronic or persistent hypoxia is a complex process involving both heme proteins [such as mitochondrial electron transport chain (ETC) complex III or IV and NADPH oxidase subtypes] and non-heme proteins [such as Proline hydroxylase (PHD) or heme oxygenase] (Taylor and Colgan, 2017). Once hypoxia is detected, cells adapt by adjusting the activities of the mitochondrial ETC and tricarboxylic acid cycle (TCA) cycle (Kumar and Choi, 2015). Hypoxia inhibits mitochondrial aerobic respiration, activates glycolysis and non-aerobic production of ATP, while also promoting erythropoiesis and angiogenesis to improve the oxygen-carrying capacity of blood and its distribution in tissues (Kumar and Choi, 2015). It is worth noting that HIFs are central to the process of cellular sensing and adaptation to hypoxia (Semenza, 1999). Clinical studies and animal models have confirmed the role of HIFs as core transcription factors regulating oxygen homeostasis in hypoxic pulmonary hypertension (HPH). A growing body of evidence suggests that HPH leads to hypoxia-dependent mitochondrial changes. Notably, There are two kinds of hypoxia that can be induced by physiologically or pathologically.

## 2 Types of hypoxia and crosstalk with mitochondria

### 2.1 Physiological hypoxia

The oxygen level can vary greatly within cells of different organs or tissues, ranging from 1% to 13% in what is known as a physical gradient (Kumar and Choi, 2015; Taylor and Colgan, 2017), such as in the juxtaposition of the intestinal mucosal surface with anoxic intestinal lumen (Albenberg et al., 2014; Glover et al., 2016), also in the renal medulla, necessary for maintaining renal function and erythropoietin (EPO) synthesis in the lymph node germinal center after B type cell numbers increased (Carreau et al., 2011; Haase, 2013; Cho et al., 2016; Wang et al., 2022a), as well as in bone marrow tissue supports the self-renewal capacity of hematopoietic stem cells (Hermitte et al., 2006; Eliasson et al., 2010; Spencer et al., 2014). Special conditions, such as in the growth and development, exercise, underwater diving, or high altitude, all of that can lead to tissue hypoxia like in skeleton muscle for the demanding oxygen of metabolic requirements after heavy sports activities (Murray et al., 2018; Wu et al., 2020; Lemieux and Birot, 2021; Mulder et al., 2021).

### 2.2 Pathological hypoxia

Patients who have been infected by pathogens such as bacteria, fungi and viruses [including severe acute respiratory syndromes-corona virus-2 (SARS-CoV-2)], the virus that causes corona virus disease-19 (COVID-19) may experience impaired oxygen exchange and progressively worsening hypoxemia (Werth et al., 2010; Friedrich et al., 2017; Devraj et al., 2017; Cavezzi et al., 2020). This can be worsened by bacterial infections due to the infiltration of inflammatory cells, the proliferation of different cell types and the activation of oxygen-consuming enzymes (Colgan et al., 2016; Serebrovska et al., 2020; Rahman et al., 2021). Meanwhile, hypoxic microenvironments form in solid tumors when the oxygen demand of rapidly growing tumor cells exceeds the supply of surrounding blood vessels. This triggers increased production of pro-angiogenic factors in response to hypoxic stimulation, promoting tumor angiogenesis and growth (Wigerup et al., 2016; Ou and Lv, 2020). However, poor neovascularization can lead to increased hypoxia and hypoxia-generated intratumoral oxygen gradients promote tumor plasticity and heterogeneity (Popper, 2016; Arpino et al., 2017; Garcia-Bermudez et al., 2018; Jing et al., 2019). Ischemia and tissue hypoxia are closely related and often occur in diseases like pulmonary infarction and myocardial infarction, where tissue hypoxia is caused by an imbalance in oxygen supply and demand (Zhang et al., 2018a). Chronic intermittent hypoxia, observed in obstructive sleep apnea syndrome, is an abnormal cycle of hypoxia and reoxygenation caused by recurrent episodes of upper airway collapse during sleep (Vicente et al., 2016). Rapid reoxygenation of transient ischemic tissues can lead to the release of reactive oxygen species (ROS), worsening tissue injury (Lam et al., 2012). Chronic lung diseases, such as COPD and pulmonary interstitial fibrosis, present with hypoxia due to impaired gas exchange in the lungs. This can worsen the disease and promote disease progression with comorbidities such as pulmonary hypertension (PH) and pulmonary heart disease (Della Rocca et al., 2022).

### 2.3 Mitochondrial monitoring for cellular sensing of both acute and long-term hypoxia

The acute hypoxia sensing mechanism takes place primarily in the carotid body, which is a hyperperfusion structure located at the bifurcation of the carotid artery. It has been reported that mitochondrial complex I signaling is altered in Glomus cells from carotid body under hypoxia. This alteration leads to accumulate NADH and ROS, change activities of the membrane ion channels, downregulate O_2_-sensitive K^+^ channels (Yamamoto et al., 2006; Fernández-Agüera et al., 2015; Arias-Mayenco et al., 2018), which trigger depolarization and cytoplasmic calcium inward flow, causing the release of neurotransmitters such as dopamine and norepinephrine (Gao et al., 2017; Arias-Mayenco et al., 2018). These neurotransmitters stimulate afferent fibers in the respiratory and autonomic centers of the brainstem, leading to deeper and faster respiration and increased cardiac output (Ortega-Sáenz et al., 2003; Ortega-Sáenz and López-Barneo, 2020; López-Barneo and Ortega-Sáenz, 2022). It is intriguing that the earlier studies have shown that inhibition of the mitochondrial electron transport chain by using mitochondrial inhibitors such as rotenone (complex I), malonate (complex II), antimycin A (complex III), and sodium azide (complex IV) increases carotid body perception and ventilatory response (Ortega-Sáenz et al., 2003; Wyatt and Buckler, 2004). This supports the “mitochondrial metabolism hypothesis” of carotid body oxygen perception. Interestingly, Moreno-Domínguez et al. (2020) revealed that genetic inactivation of mitochondrial complex IV subunit type 2 (Cox4i2) or Epas1 largely abolished the hypoxic response. Moreover, expression of Cox4i2, Ndufa4l2 (complex I subunit), and Cox8b (complex IV subunit) was significantly reduced in mice approximately 2 months after Epas1 gene inactivation. Further studies revealed that hypoxia-induced increase in mitochondrial NADH and ROS were abrogated in Cox4i2 knockout mice (Moreno-Domínguez et al., 2020). The study proposed the novel idea that complex IV acts as an oxygen sensor under hypoxia, causing electron backlogging in the ETC and accumulation of reduced coenzyme Q (QH2). This leads to a decrease in the function of complex I, the main effector of hypoxic mitochondria, which in turn leads to an increase in the production of signaling molecules (NADH and ROS) that regulate ion channels. Although the details of this process require further investigation, the study sheds light on the presumed process of acute hypoxia perception in the carotid body. It also identifies an unexpected role for HIF2α in linking acute and chronic hypoxic adaptive response.

Chronic hypoxia sensing is more complex, and its mechanism is not yet fully understood. The PHD/HIFs oxygen sensing pathway axis has been extensively studied and plays a critical role in regulating cell hypoxia adaptation (Semenza, 2012). PHD belong to the α-ketoglutarate-dependent dioxygenase superfamily, which hydroxylates proline residues in HIFs under normoxic conditions. Hydroxylated HIFs are degraded by ubiquitin proteases, but under hypoxic conditions, PHD activity is inhibited, allowing HIFs to avoid degradation, form heterodimers, and regulate target gene expression (Metzen and Ratcliffe, 2004; Kaelin, 2005). This promotes cell adaptation to hypoxia and maintains oxygen homeostasis (Prabhakar and Semenza, 2012). PHD-independent cellular hypoxia perception also occurs through other proteins in the α-ketoglutarate-dependent dioxygenase family, such as collagen proline 4-hydroxylases, histone lysine demethylases containing the Jumonji domain (JmjC-KDMs), and the 10–11 translocation enzyme (TET) family (Kaelin, 2005; Markolovic et al., 2015). These proteins can sense hypoxia and reshape the transcriptional activity of HIFs, leading to structural and/or functional damage to mitochondria.

**FIGURE 1:**
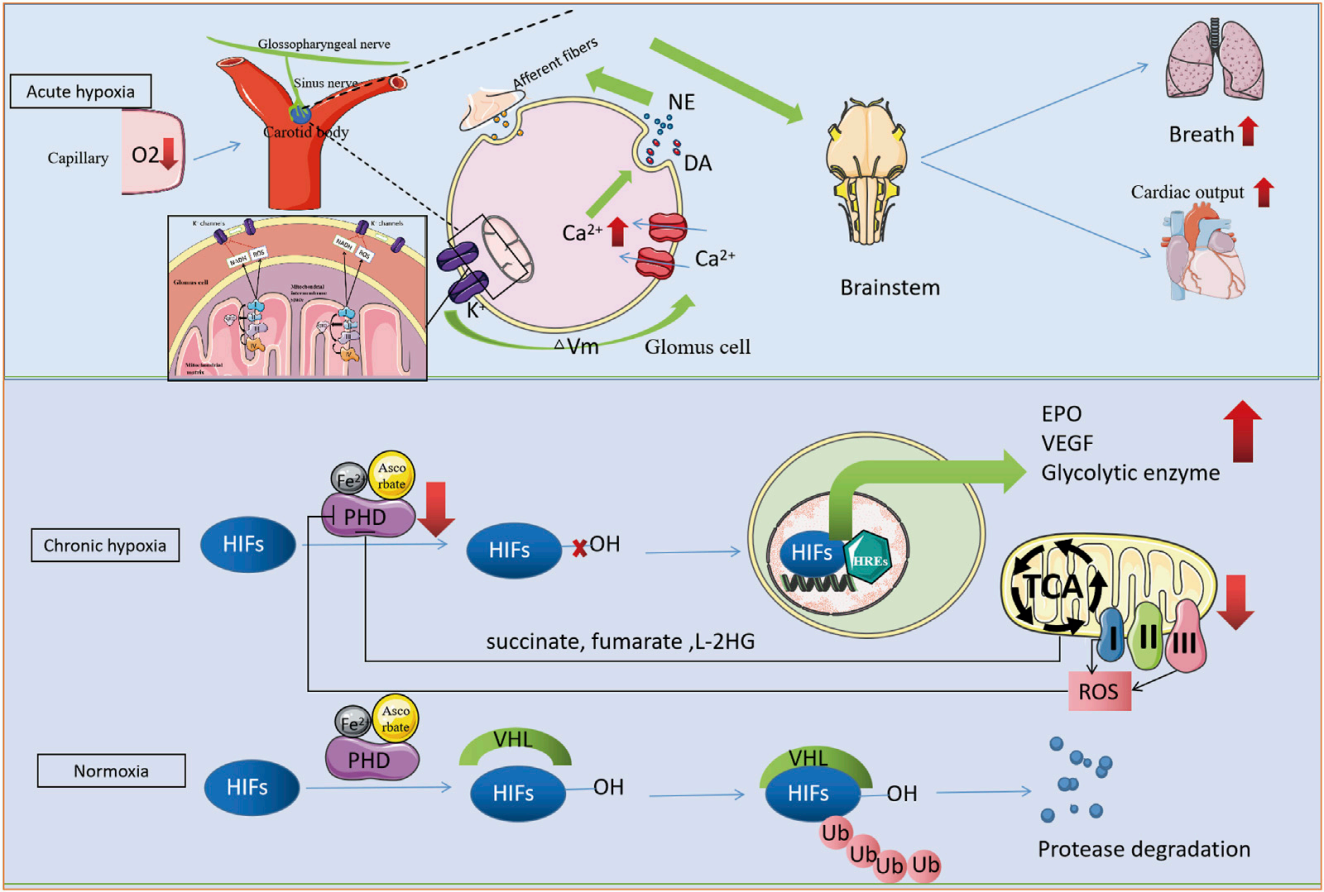
Hypoxic perception and mitochondrial interaction in cells. Acute hypoxia is detected by the Glomus cell of the carotid body as a decrease in capillary blood oxygen content, which leads to accumulate NADH and ROS from complex I, downregulate O_2_-sensitive K^+^ channels and a decrease in the potential difference between the inside and outside of the cell membrane (∆Vm). This depolarizes the cell membrane, allowing an inward flow of calcium ions and the release of neurotransmitters such as dopamine and norepinephrine. These neurotransmitters stimulate the afferent fibers in the respiratory and autonomic centers of the brainstem, leading to accelerated respiratory deepening and increased cardiac output. Chronic hypoxia, on the other hand, is detected by PHD/HIFs. Under normoxia, PHD hydroxylates HIFs, which are then recognized and bound by VHL, leading to HIFs ubiquitin protease degradation. Under hypoxia, PHD activity is reduced or even inactivated, and HIFs are spared from degradation and translocated to the nucleus. There, they bind to hypoxic response elements (HREs) that regulate gene expression related to cell metabolism, such as glycolysis and oxidative phosphorylation (OXPHOS). TCA cycle intermediates and oxidative phosphorylation by-products ROS further regulate PHD activity, thus forming a closed loop of negative feedback.

Interestingly, metabolites produced by the mitochondrial TCA, such as succinate, fumarate, and L-2-hydroxyglutarate (L-2HG), as well as ROS regulate the α-ketoglutarate-dependent dioxygenase family (Bargiela et al., 2018; Lee et al., 2020). The interaction between hypoxia and mitochondria through the α-ketoglutarate-dependent dioxygenase family suggests that interventions targeting this family could potentially prevent hypoxia-induced damage to mitochondria (Figure 1).

## 3 The role of hypoxia-induced mitochondrial dysregulation in HPH

Mitochondria are crucial for maintaining cellular metabolic functions. They provide energy in the form of ATP through the ETC and act as a metabolic hub through the TCA cycle. Additionally, by producing ROS as intracellular signals, they regulate various biological functions (Annesley and Fisher, 2019; Bao et al., 2021). However, when cells are exposed to hypoxia, mitochondrial homeostasis is disrupted, resulting in structural and functional changes that are essential for the cells to sense and adapt to hypoxia. A growing body of evidence suggests that hypoxia-induced mitochondrial dysregulation play an important role in the development of HPH.

The hallmark of HPH is a mean pulmonary artery pressure greater than 25 mmHg at rest, leading to severe progressive lung disease, right heart failure, and premature death due to exposure to hypoxia (Luna-López et al., 2022). The World Health Organization (WHO) classifies PH into five clinical subtypes based on etiology. HPH, the third subtype, is often associated with chronic lung diseases such as COPD, interstitial pulmonary fibrosis, sleep apnea syndrome, or long-term residence in high-altitude hypoxic areas. The 1-year survival rate for PH ranges from 68% to 93%, while the 3-year survival rate is between 39% and 77%, according to the PH Registry (Galiè et al., 2016; Simonneau et al., 2019; Vachiéry et al., 2019). However, a recent cohort study showed that HPH due to chronic lung disease or hypoxia had a 1-year, 3-year, and 5-year survival rate of 79%, 48%, and 31%, respectively (Rose et al., 2019). Patients with more severe lung disease and severe PH have a worse prognosis. Patients with HPH have lower survival rates, as clinical investigations have shown that HPH significantly correlates with patients’ functional decline and disease deterioration (Seeger et al., 2013; Hayes et al., 2016; Medrek et al., 2017). While the precise pathogenesis of HPH requires further exploration, its key features include hypoxic pulmonary vasoconstriction (HPV) and pulmonary vascular remodeling. Alveolar hypoxia constricts intrapulmonary arteries, a protective mechanism known as HPV, which optimizes gas exchange by adapting blood flow to alveolar ventilation (Dunham-Snary et al., 2017). Mitochondria act as oxygen sensors, altering cytoplasmic redox status by affecting mtROS production under hypoxia, which in turn regulates HPV mediators (e.g.,: ion channels and kinases) (Sommer et al., 2016). Chronic hypoxia and persistent HPV can result in pulmonary vascular remodeling, increased pulmonary vascular resistance and PH.

### 3.1 Mitochondria and HPV

HPV has been extensively studied, but the molecular mechanism behind it is not yet fully understood (Waypa and Schumacker, 2008; Sylvester et al., 2012). Changes in mitochondrial ROS production in pulmonary arterial smooth muscle cells (PASMCs) are considered to be the most likely mechanism to trigger HPV. However, whether hypoxia causes an increase or a decrease in mitochondrial ROS production remains controversial. The initial theory suggested that ROS production in the electron transport chain decreased due to a reduction in substrate during hypoxia. Archer et al. (1989) confirmed this conclusion by detecting ROS fluorescence probe in pulmonary artery circulation. On the contrary, more recent studies have confirmed an increase in mitochondrial ROS production during hypoxia (Jefferson et al., 2004; Desireddi et al., 2010; Sommer et al., 2010; Pak et al., 2018), especially superoxide, which is rapidly converted by superoxide dismutase 2 (SOD2) to hydrogen peroxide. The latter acts as a diffusible redox mediator and regulates redox-sensitive ion channels, such as potassium and calcium channels.

The voltage-gated potassium channels (Kv) on PASMCs maintain a resting membrane potential of approximately −60 mV. This negative membrane potential inhibits the opening of voltage-gated L-type calcium channels. During hypoxia, the outward potassium current is inhibited, the membrane is depolarized, the calcium channel is opened, calcium flows in, the cytoplasmic calcium increases, and then Rho kinase-mediated calcium sensitization leads to the contraction of the pulmonary artery (Wang et al., 2004; Desireddi et al., 2010). The source of the hypoxia-induced increase in mitochondrial ROS is primarily complex III (Leach et al., 2001; Guzy et al., 2005; Waypa et al., 2006; Waypa et al., 2013). However, it has been shown that inhibition of either complex I or III attenuates hypoxia-induced HPV (Waypa et al., 2001; Dunham-Snary et al., 2019). Studies also showed that genetic deletion of Rieske iron-sulfur protein (RISP, complex III cofactor) in PASMCs counteracts hypoxia-induced increase in mitochondrial ROS and cytoplasmic calcium (Guzy et al., 2005; Waypa et al., 2013). This highlights the importance of mitochondrial complex III in HPV oxygen sensing.

### 3.2 Hypoxia impaired mitochondrial homeostasis in pulmonary vascular cells

Persistent HPV can lead to pulmonary vascular remodeling and the subsequent development of pulmonary hypertension (PH). Physiologically, the thickness of the pulmonary vessel wall is maintained by a balance of cell proliferation and apoptosis. However, under hypoxia, this balance is disrupted, and pulmonary vascular cells [including pulmonary artery endothelial cells (PAECs), PASMCs and fibroblasts] exhibit a phenotype of high proliferation and resistance to apoptosis. This leads to thickening of the pulmonary vascular wall and obstruction of the vascular lumen, a process of structural remodeling known as hypoxic pulmonary vascular remodeling (Crosswhite and Sun, 2014). Moreover, hypoxia also disrupts mitochondrial homeostasis in pulmonary vascular cells, which plays an important role in hypoxic pulmonary vascular remodeling, particularly in PASMCs. While this has been extensively studied in PASMCs, it is also important to investigate its impact on PAECs and fibroblasts.

#### 3.2.1 PASMCs

A chronic swift in energy metabolism from OXPHOS to glycolysis can be observed in human and rodent hypoxia-treated PASMCs (Rafikov et al., 2015; Parra et al., 2017; Lu et al., 2021; Ma et al., 2022), that also known as the “ Warburg effect,” initially described in tumor cells, lead to a highly proliferative and apoptosis-resistant phenotype in PASMCs. The increased expression of NADH dehydrogenase (ubiquinone) 1 alpha subcomplex 4 like 2 (NDUFA4L2), an important subunit of mitochondrial complex I in hypoxia, is attributed to this metabolic switch. This leads to decreased complex I activity, increased mitochondrial ROS production, activation of HIF1α, and promotion of glycolysis-related proteins such as lactic dehydrogenase (LDH) and pyruvate dehydrogenase kinase 4 (PDK4) expression, as well as decreased expression of mitochondrial OXPHOS-related proteins such as PDH (Liu et al., 2021; Liu et al., 2022; Li et al., 2023). Ultimately, this results in excessive proliferation of PASMCs.

Additionally, mitochondrial phenotypes such as morphology change with hypoxia. PASMCs from human and rodent PH model sources showed an increase in mitochondrial number and smaller size after hypoxia treatment. This change is characterized by mitochondrial network fragmentation and an increase in membrane potential (Xu et al., 2016). Furthermore, hypoxia increased the mitochondrial fission and mitophagy of PASMCs, which aggravated the endoplasmic reticulum pressure and contributed to the excessive proliferation of PASMCs (Zhu et al., 2017; Parra et al., 2017; Liu et al., 2022; Zhuan et al., 2020). Interestingly, there is a crosstalk between mitochondria and endoplasmic reticulum. Under hypoxia, increased mitochondrial ROS in PASMCs leads to an increase in calcium release via ryanodine receptor-2 (RyR2) on the endoplasmic reticulum and an increase in the mitochondrial calcium unidirectional transporter (MCU). This subsequently induces a further increase in RISP-dependent mitochondrial ROS, creating a positive feedback between mitochondria and the endoplasmic reticulum (Yang et al., 2020). Moreover, hypoxia also induces increased expression of neurite outgrowth inhibitor-B (Nogo-B) on PASMCs, which acts as a regulator of endoplasmic reticulum structure. This increases the distance between the ER and mitochondria, reduces the phospholipid transfer from the ER to the mitochondria, and decreases mitochondrial calcium and pyruvate dehydrogenase (PDH) activity. The reduction and suppression of cellular oxidative metabolism result in a glycolytic metabolic phenotype, excessive proliferation, and resistance to apoptosis of PASMCs (Sutendra et al., 2011; Dromparis et al., 2013).

#### 3.2.2 PAECs

Although the mitochondrial alterations in PAECs induced by hypoxia have been less well studied, mitochondrial disorders have been reported in the presence of PAECs. Hypoxia induces altered mitochondrial morphology, characterized by mitochondrial swelling and cristae loss, in endothelial cells of hypoxia-induced PH rats. Hypoxia also reduces peroxisome proliferator-activated receptor coactivator (PGC-1) expression in PAECs, inhibits ATP production, increases mitochondrial ROS formation, and induces mitochondrial fragmentation, resulting in endothelial cell dysfunction (Ye et al., 2016; Suresh et al., 2019).

#### 3.2.3 Fibroblasts

HPH models and human fibroblasts exhibit downregulation of NDUFS4, an auxiliary subunit of mitochondrial complex I. This leads to reduced complex I activity, increased mitochondrial ROS production, mitochondrial fragmentation, and reduced PDH activity, resulting in suppressed mitochondrial bioenergy. These effects contribute to a hyperproliferative, anti-apoptotic phenotype of fibroblasts (Plecitá-Hlavatá et al., 2016).

### 3.3 Distinctive alterations of mitochondria from cells in HPH

Hypoxia can induce changes in mitochondrial number, size, and ultrastructure globally, which depend on the severity and duration of exposure periods (Mironova et al., 2019; Quintana et al., 2019; Lukyanova et al., 2021). Such as In PASMCs, hypoxia increased the number of mitochondria while reducing their size. In contrast, the mitochondria of PAECs showed swelling and cristae loss (Xu et al., 2016; Ye et al., 2016). These changes can affect mitochondrial dynamics, leading to reduced fusion and an increased tendency towards fission, resulting in smaller or fragmented mitochondria (Xu et al., 2016; Ye et al., 2016; Suresh et al., 2019). Interestingly, chronic intermittent hypoxia can activate the AMPK-PGC1α-Sirt3 signaling pathway, promoting mitochondrial biosynthesis, repairing mitochondrial ultrastructural damage, and improving mitochondrial function (Su et al., 2022). Mitochondrial biosynthesis is a complex process that requires coordinated efforts between mitochondrial DNA (mtDNA) and nuclear DNA, regulated by PGC1α and nuclear respiration factors (NRFs) (Wenz, 2013). Hypoxia can disrupt mitochondrial biosynthesis by affecting PGC1α activity in a cell-specific manner (Zhu et al., 2010; Tohme et al., 2017; Qian et al., 2019; Wang et al., 2022b). However, the role of mitochondrial biosynthesis in HPH needs to be explored in depth.

#### 3.3.1 Mitochondrial dynamic imbalance and HPH

Mitochondria are organelles that are constantly undergoing fusion and fission events in order to maintain their structure and function. The balance between fission and fusion proteins regulates these events and changes in their expression levels can result in morphological changes in mitochondria (Hara et al., 2018; Zemirli et al., 2018). Hypoxia is a critical factor in maintaining the balance between mitochondrial fusion and fission. Several studies indicate that hypoxia can increase mitochondrial fission in various cell types by upregulating Dynamin-related protein 1 (Drp1) activity through HIF1α (Table 1). However, some research suggests that hypoxia can also promote mitochondrial fusion in certain cells by increasing the activity of mitofusin (MFNs) and optic atrophy (OPA) (Wang et al., 2020; Chiche et al., 2010). Therefore, the effects of hypoxia on mitochondrial dynamics may depend on the cell type (Table 1).

**TABLE 1:** Imbalance mitochondrial dynamics under hypoxia.

Mitochondrial dynamics	Cell types	Involved in pathways	Diseases	References
Increasing fisson	Pancreatic beta cell	HIF-1α/pDRP1 (Ser616)	Diabetes	Zhang et al. (2018b)
	Neurons	unkonwn	Neurodegeneration	Jain et al. (2015)
	Brain microvascular endothelial cells	Drp1	Dementia	Chen et al. (2017)
	PASMCs	Drp1 Aldehyde dehydrogenase 2 (ALDH2)/HIF-1α/pDRP1(Ser616) HIF-1α/DRP1	PH	Zhuan et al. (2020), Parra et al. (2017), Zhao et al. (2019), Chen et al. (2019), Chiche et al. (2010)
			HPH	
			PH	
	HK-2cell	HIF-1α/haeme oxygenase-1(HO-1)	Diabetic nephropathy	Vangrieken et al. (2021)
	HeLa and HEK293T cells	USP19/FUNDC1/Drp1	Mitochondrial dynamics in response to hypoxia	Chai et al. (2021)
	ovarian cancer cells	Salt-Inducible Kinase (SIK2)/Drp1 ROS/p-Drp1 (Ser637)/MFN1	Ovarian cancer	Gao et al. (2020), Han et al. (2019)
	Head and neck squamous cells	ROS/HIF-1α/MFF	head and neck squamous cell carcinoma	Wu et al. (2021a)
	hepatocellular carcinoma	DRP1 HIF-1α/NDRG1(N-myc downstream-regulated gene-1)/Drp1/Fis1	hepatocellular carcinoma	Lin et al. (2020), Guo et al. (2020a)
	HEK293 cells	extracellular signal-regulated kinase (ERK)/Drp1	Alzheimer’s disease	Yuan et al. (2021)
	Endothelial progenitor cells	pDRP1 (Ser637)/Fis1/MFN1/OPA1	Cancer	Kim et al. (2018)
	BeWo cells	ROS?	Impaired utero-placental perfusion	Vangrieken et al. (2021)
	Placental mesenchymal stromal cells	HIF-1α/DRP1	Preeclampsia	Gillmore et al. (2022)
	Cardiomyocytes	Haemin/HO-1 hypoxia inducible domain family member 1B (HIGD-1B)/OPA-1	intermittent hypoxia-induced cardiac injury, Heart disease associated with hypoxia	Han et al. (2018), Marsboom et al. (2012)
	Glioma cells	HIF-1α/HCLS1-associated protein X-1 (HAX1)/pAKT/Drp	Glioma	Linqing et al. (2021)
Increasing fusion	Bone marrow mesenchymal stem cells	OPA1, PINK1, and Parkin	Wound healing	Wang et al. (2020)
	PC3/LS174 cells	MFN1,BNIP3/BNIP3L	Cancer	Chiche et al. (2010)

Mitochondrial dynamics including the processes of fusion and fission, are essential for maintaining mitochondrial function and cellular homeostasis. Hypoxia can affect these dynamics and, in turn, alter mitochondrial function. Under hypoxia, mitochondria fusion or fission can be induced in a cell type-specific manner. This process merges two or more mitochondria into a single organelle, mainly regulated by the proteins MFN1, MFN2, and OPA1. Increased fusion promotes the exchange of mitochondrial contents, such as mtDNA and proteins, and helps maintain mitochondrial function under stress conditions (Rambold et al., 2011). HIF1α induces BNIP3 and BNIP3L/NIX expression, which promotes mitochondrial fusion by inactivating Drp1 and promoting OPA1 processing. Meanwhile, mitochondrial fission divides a single mitochondrion into two or more smaller organelles. Drp1 and its adaptor proteins—such as mitochondrial fission 1 protein (Fis1), mitochondrial fission factor (MFF), and mitochondrial dynamics proteins of 49 and 51 kDa (MiD49/51) primarily regulate this process. Fission is essential for removing damaged mitochondrial components and facilitating mitophagy (a selective degradation of mitochondria by autophagy) to maintain mitochondrial quality (Pickles et al., 2018). Hypoxia also induces mitochondrial fission through HIF1α-mediated upregulation of Drp1, leading to increased mitochondrial fragmentation (See Table 1 for details).

Previous studies indicate that a dysregulation of mitochondrial fission and fusion takes place in patients with PH and experimental animal models. The most common imbalance observed is an excess of fission over fusion, resulting in mitochondrial fragment. This structural change is characterized by an increase in the expression and activity of fission factors, in particular DRP-1, and a decrease in the expression of fusion factors, mainly MFN2. The pathophysiology of HPH is characterized by the dysfunction of PAECs, excessive proliferation, and resistance to apoptosis of PASMCs and fibroblasts. The imbalance of mitochondrial fission and fusion leads to excessive proliferation, resistance to apoptosis, increased migration, and a metabolic phenotypic switch of pulmonary artery vascular cells (Rambold et al., 2011; Hong et al., 2017; Parra et al., 2017). Mitochondrial fission plays a crucial role in the pathogenesis of HPH. The increased expression levels and/or activity of Drp1 and its adapter proteins due to epigenetic regulation, transcriptional regulation, and post-translational modifications primarily result in mitochondrial fission (Chen et al., 2018a; Wu et al., 2021a). In mitochondrial fission, the endoplasmic reticulum wraps around mitochondria, marking the fission site. Cytoplasmic Drp1 is activated through post-translational modifications and moves to the outer mitochondrial membrane, where adapter proteins such as MiD49, MiD51, MFF, and Fis1 form a ring-like structure that contracts the mitochondrial membrane using energy from GTP hydrolysis, leading to fission (Chen et al., 2019). Post-translational modifications of Drp1, such as phosphorylation at the serine S616 site, promote mitochondrial fission, while dephosphorylation at the serine S637 site reduces it. These modifications activate Drp1, causing a hyperproliferative and anti-apoptotic phenotype in PASMCs. HIF-1α, under hypoxia, is the primary transcription factor regulating Drp1 activity by modulating the Drp1 promoter hypoxia response element (Xiao et al., 2022a). Other molecules that regulate Drp1 activity, such as cyclin B1/cyclin-dependent kinase (CDK1), contribute to the pathogenesis of HPH by triggering PASMCs proliferation and PH development (Feng et al., 2021).

The use of a Drp1 inhibitor, mdivi-1, has been shown to reduce hypoxia-induced fragmented mitochondrial morphology in PASMCs, decrease endoplasmic reticulum stress, inhibit PASMCs proliferation, and alleviate hypoxia-induced pulmonary hypertension and vascular remodeling (Zhuan et al., 2020; Chen et al., 2018a; Xiao et al., 2022b). Increased expression of MiD49 and MiD51, Drp1 adapter proteins, has been observed in PH patients and hypoxia-treated PASMCs, promoting mitochondrial fission by recruiting Drp1 to the outer mitochondrial membrane and activating extracellular regulated protein kinases (ERK1/2), which leads to Drp1 phosphorylation at the serine 616 site (Chen et al., 2018b; Xiao et al., 2022a). This triggers mitophagy, bone morphogenetic protein receptor 2 (BMPR2) lysosomal degradation, and inhibition of DNA binding (Id1) downregulation, increasing PASMCs proliferation and migration (Feng et al., 2021).

The mitochondrial fission in PH is regulated epigenetically. In the circulation of PH patients and hypoxia-induced animal models, decreased expression of miR-344a-3p has been observed. Additionally, according to Chen et al. (2018a), downregulation of miR-34a-3p in pulmonary artery smooth muscle cells (PASMCs) from a hypoxia-induced pulmonary hypertension (PH) rat model upregulates MiD49 and MiD51. These proteins regulate DRP1-mediated mitochondrial fission, leading to the emergence of a highly proliferative and anti-apoptotic PASMC phenotype. The essential Drp1 adapter protein MFF is also increased in hypoxia-treated PASMCs, and MFF knockdown has been shown to inhibit PASMCs proliferation and induce apoptosis by negatively regulating SIRT1/3 expression. Silencing MFF has been found to improve hypoxic PAMSCs mitochondrial function by increasing ATP production, reducing ROS production, and decreasing mitochondrial fission. MFF is the target of miR-340-5p, which negatively regulates MFF expression in PAMSCs under hypoxia (Huang et al., 2022). Mitochondrial dynamic imbalance in PAECs is also crucial for the development of HPH. Hypoxia has been found to promote Ca^2+^-dependent proliferation and migration of PAECs and prevent apoptosis by inducing Drp1 activation and increased mitochondrial fission (Shen et al., 2015).

#### 3.3.2 Mitochondrial fusion and HPH

**FIGURE 2:**
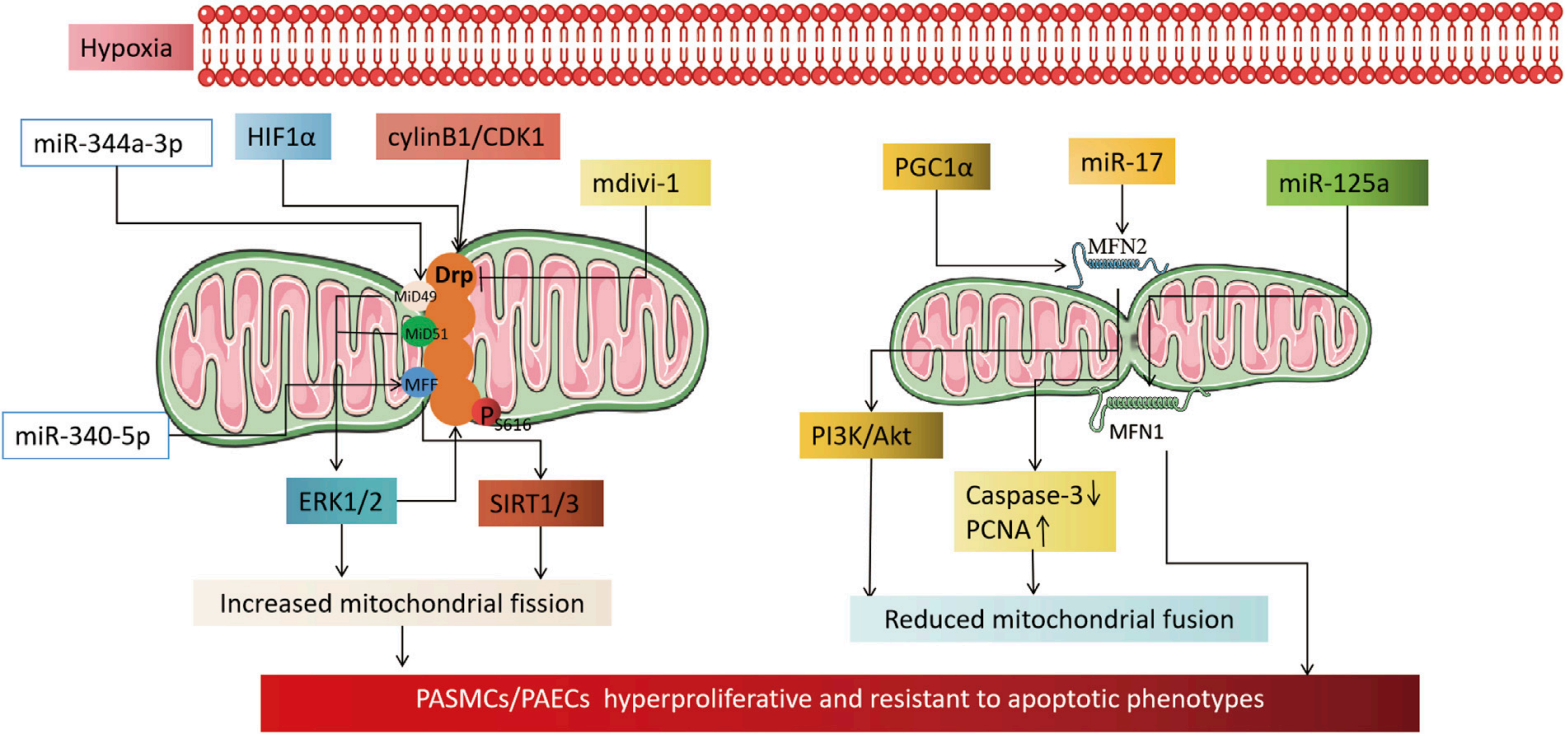
Mechanism by which hypoxia causes pulmonary hypertension by affecting mitochondrial dynamics. On the left, it illustrates the mechanism by which an increase in mitochondrial fission, induced by hypoxia, leads to an altered phenotype of pulmonary artery vascular wall cells. The activation of HIF1α under hypoxia transcriptionally regulates Drp1 activity by modulating the hypoxia response element of the Drp1 promoter. CylinB1/CDK1 also plays a role in regulating Drp1 activity. The application of Drp1 inhibitor mdivi-1 can reduce the proliferative, anti-apoptotic phenotype of pulmonary vascular wall cells by reducing mitochondrial fission. Epigenetic miR-344a-3p and miR-340-5p regulate the activity of DRP1 adaptor proteins MiD49/51 and MFF. miR-138 and miR-25 downregulate the mitochondrial calcium unidirectional transporter (MCU), which leads to increased mitochondrial fission by increasing the expression of mitochondrial network factor (MNF). The right part shows that a reduction in mitochondrial fusion, induced by hypoxia, is involved in the mechanism of cellular phenotypic alterations in the pulmonary artery vessel wall. PGC1α and miR17 downregulate MFN2 under hypoxia, which reduces mitochondrial fusion through the PI3K/Akt pathway, downregulating Caspase3, and upregulating PCNA. Whereas MFN1 is committed to the development of pulmonary hypertension, miR-125a agonist alleviates the promoting effect of MFN1 on pulmonary vascular remodeling.

MFN, a key factor that helps fuse mitochondrial outer membranes, is inhibited by hypoxia. This inhibition results in MFN2 proteasomal degradation and decreased expression, which in turn promotes the proliferation of pulmonary artery smooth muscle cells and inhibits their apoptosis (Dasgupta et al., 2021; Chen et al., 2022; Zhu et al., 2017). Conversely, increasing MFN2 can inhibit PASMCs proliferation, induce apoptosis, and alleviate hypoxia-induced pulmonary hypertension in rats (Ryan et al., 2013). PGC1α, a transcriptional coactivator of MFN2, mediates MFN2 downregulation under hypoxia (Ryan et al., 2013). It activates the PI3K/Akt pathway, leading to mitochondrial fragmentation and increased PASMCs in the S + G2/M phase of the cell cycle. Moreover, it inhibits the mitochondrial apoptotic pathway, triggering a proliferation-apoptosis imbalance in PASMCs (Ryan et al., 2013; Zhang et al., 2012). Epigenetics also plays a role in regulating MFN2 activity. In hypoxia-treated PASMCs, miR-17 expression is upregulated. miR-17 inhibits MFN2 expression by binding to its 3′-untranslated region, leading to downregulation of cleaved caspase-3 expression and upregulation of proliferating cell nuclear antigen (PCNA) expression, resulting in a highly proliferative, anti-apoptotic phenotype in PASMCs (Lu et al., 2016). Unlike MFN2, MFN1 may contribute to the development of PH. MFN1 expression increases in hypoxia-treated PASMCs and mouse models of PH, while miR-125a agonists alleviate MFN1’s promotional effect on pulmonary vascular remodeling (Ma et al., 2017). Imbalanced mitochondrial fission/fusion under hypoxia leads to excessive mitochondrial fragmentation, which triggers PH. The primary cause is the upregulation of fission-related genes (Drp1 and adapter proteins MiD49, MiD51, MFF, and MNF) or downregulation of fusion-related genes (MFN2). The fission/fusion imbalance results in PAECs dysfunction, PASMCs hyperproliferation, and anti-apoptotic phenotypes. As such, it could be a potential therapeutic target for HPH (Figure 2).

#### 3.3.3 Adaptive mitophagy in HPH

Mitophagy, the selective autophagy degradation of damaged mitochondria, is an essential process for maintaining mitochondrial quality control. The PTEN-induced putative protein kinase 1/E3 ubiquitin ligase PARK2 (PINK1/Parkin) system plays a significant role in mitochondrial non-receptor-mediated autophagy (Pickles et al., 2018). Activation of the PINK1-Parkin pathway by HIF-1α under hypoxia induces mitophagy, protecting cells from hypoxia-induced apoptosis and ROS generation (Li et al., 2020; Yu et al., 2022).

**FIGURE 3:**
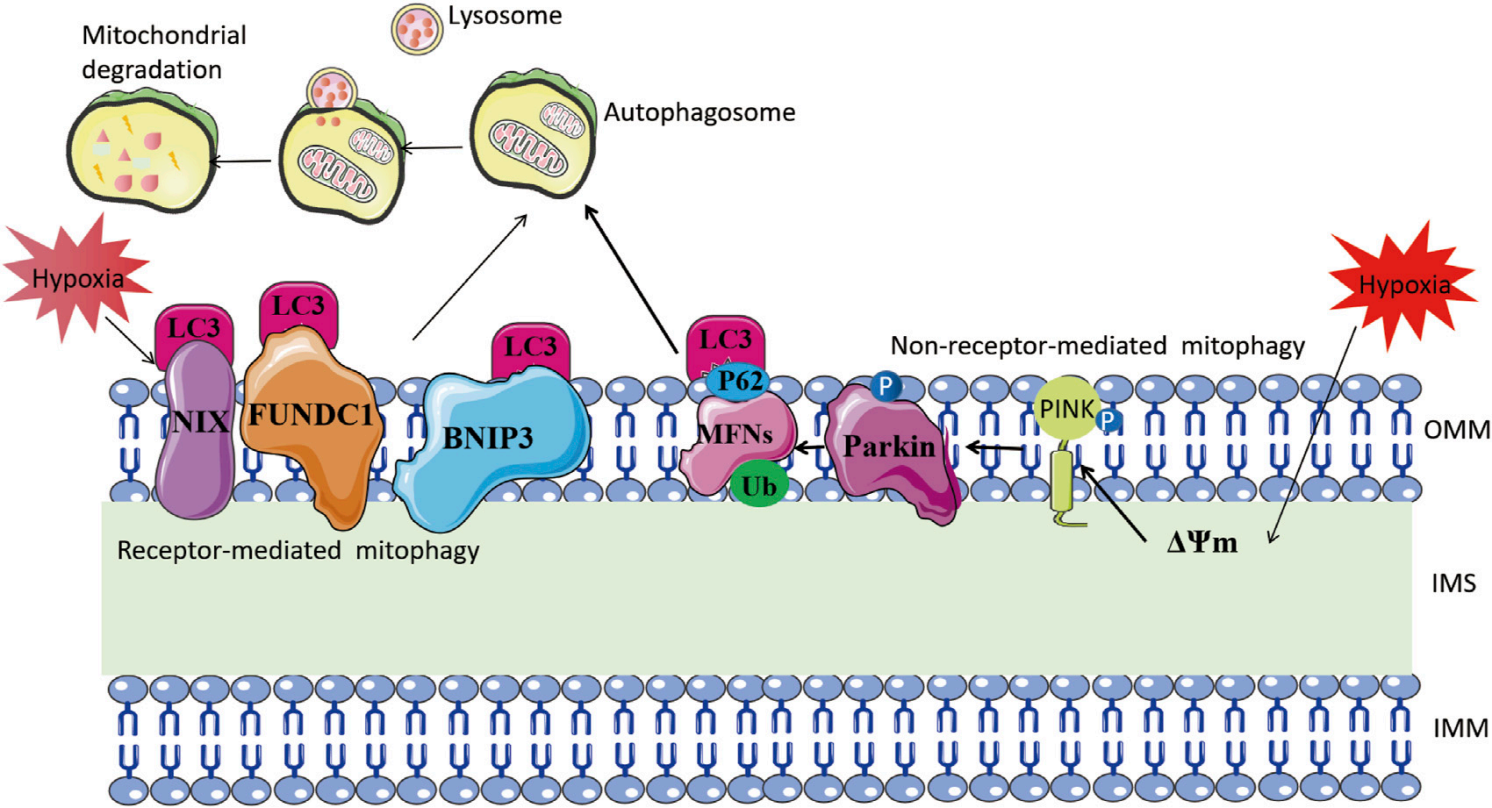
Mitophagy mechanism under hypoxia. The left part illustrates receptor-mediated mitochondrial autophagy. Hypoxia stimulates mitochondrial outer membrane proteins such as BNIP3, NIX, and FUNDC1 to bind directly to LC-3 through their LC3-interacting region motifs. This, in turn, forms an autophagosome and leads to mitochondrial degradation. The right side shows non-receptor-mediated mitochondrial autophagy. Hypoxia induces mitochondrial membrane depolarization, which prompts the stabilization of PINK1. This, in turn, recruits Parkin to the outer mitochondrial membrane and phosphorylates it. Parkin ubiquitinates mitochondrial proteins including MFNs, and the ubiquitinated proteins are linked to LC3 via the adaptor protein SQSTM1/P62, eventually forming autophagosomes.

Receptor-dependent mitophagy mechanisms involve mitochondrial outer membrane proteins, such as BNIP3, NIX, and FUN14 domain containing 1 (FUNDC1), which act as inducible receptors upon stimuli like hypoxia and starvation (Liu et al., 2012; Madhu et al., 2020; Lampert et al., 2019) (Figure 3). Under hypoxia, HIF-1α-induced BNIP3 promotes mitophagy, protecting against renal and myocardial ischemia-reperfusion injury (Wang et al., 2023; Zhang et al., 2019; Fu et al., 2020; Yang et al., 2019). Thus, mitophagy induced by hypoxia is an adaptive metabolic response that prevents increased ROS and cell death (Zhang et al., 2008).

The process of mitophagy is implicated in the development of HPH. In animal models exposed to chronic hypoxia, PINK1 expression in PASMCs increases, leading to enhanced PINK1/Parkin-mediated mitophagy. This results in excessive proliferation and resistance to apoptosis in PASMCs, which promotes hypoxia-induced PH and pulmonary vascular remodeling (Linqing et al., 2021; Saraji et al., 2021). Interestingly, mitochondria protect PASMCs from dysfunctional mitochondria through a compensatory increase in Pink1-mediated mitophagy, which can also cause mitochondrial hyperpolarization (Saraji et al., 2021). This hyperpolarization may be associated with downregulation of uncoupling protein 2 (UCP2) and can result in a hyperproliferative, anti-apoptotic phenotype in PASMCs (Pak et al., 2013). However, downregulation or knockdown of UCP2 in endothelial cells leads to PINK1-induced excessive mitophagy and apoptosis in PAECs, ultimately contributing to intermittent hypoxia-induced PH (Haslip et al., 2015). FUNDC1, a hypoxia-induced mitophagy receptor, is involved in the process. Hypoxia induces phosphorylation of the serine 17 site of the LC3-interacting region (LIR) sequence in FUNDC1, enhancing its binding affinity to LC3 and increasing mitophagy (Wu et al., 2014; Lv et al., 2017). Mitophagy promotes PASMCs proliferation through upregulation of the ROS/HIF1α pathway, leading to pulmonary vascular remodeling and PH (Liu et al., 2022). Apoptosis-inducing factor (AIF), a mitochondrial oxidoreductase regulating mitophagy in PASMCs, is downregulated and ubiquitinated under hypoxia through interaction with ubiquitin protein UBA52, leading to increased mitophagy and PASMCs proliferation (Ma et al., 2022). In conclusion, mitophagy plays a crucial role in the development of pulmonary vascular remodeling and HPH by affecting the proliferation and apoptosis of PASMCs and PAECs. Further studies can investigate the specific roles and mechanisms of other components mediating mitophagy, such as Parkin and mitophagy receptors like BNIP3 and NIX, in hypoxia-induced PH. These studies can provide more therapeutic targets.

#### 3.3.4 Metabolic reprogramming and pulmonary vascular phenotype in HPH

Hypoxia causes diminished O_2_ levels as an electron acceptor in ETC, which leads to the releasing of ROS and activates HIF1α (Kung-Chun Chiu et al., 2019). Consequently, HIF1α promotes glycolytic capacity by increasing the expression of glucose transporter 1 (GLUT1), hexokinase 2 (HK2), PDK1, and LDH (Kim et al., 2006; Tsui et al., 2013; Kim et al., 2007; Jin et al., 2022). At the same time, HIF1α slows electron transfer in the ETC by inducing less active subunits of ETC complex I and IV, NDUFA4L2 and COX4-2, and indirectly mediating the degradation of complex I subunits NDUFB5 and NDUFB6, and complex IV subunits COX4L1 and NDUFA4 through the mitochondrial inner membrane protein OMA1. These actions downregulate OXPHOS to reduce mitochondrial oxygen consumption and adapt to hypoxia (Papandreou et al., 2006; Fukuda et al., 2007; Tello et al., 2011; Wu et al., 2021b). In addition, ETC complex I requires iron-sulfur cluster assembly assisted by iron-sulfur cluster assembly protein 1/2 (ISCU1/2), but hypoxia-induced HIF1α increases miR-210 expression, which targets ISCU1/2 mRNA and inhibits its expression, resulting in decreased complex I activity (Chan et al., 2009). Hypoxia inhibition of OXPHOS may be related to mtDNA mutation or decreased mtDNA content (Reznik et al., 2016). Interestingly, hypoxia can also increase OXPHOS in tumor cells by upregulating PGC1α in colorectal cancer cells (Yun et al., 2019). This opposite result may be attributed to PGC1α as a regulatory pathway that is not dependent on HIF1α. However, further studies are needed to fully understand the relationship between HIF1α and PGC1α under hypoxia.

Numerous studies have shown that pulmonary vascular cells (PAECs, PASMCs, fibroblasts) from animal models of hypoxia-induced PH exhibit a chronic shift from mitochondrial OXPHOS to glycolysis (Plecitá-Hlavatá et al., 2016; Xu et al., 2021; Wu et al., 2021c; Colon Hidalgo et al., 2022), this metabolic reprogramming results in PAECs dysfunction and a highly proliferative, apoptosis-resistant phenotype in PASMCs, which is associated with tumor progression and metastasis (Vaupel et al., 2019). HIFs play a central role in adaptive regulation of energy metabolism under hypoxic conditions, facilitating the conversion from mitochondrial oxidative phosphorylation to glycolysis. Under hypoxia, HIF-1α activation directly upregulates PDK1 gene expression, inhibiting PDH activity and weakening the TCA cycle (Kim et al., 2006). Additionally, HIF-1α induces miR-210 transcription in PAECs, leading to decreased expression of ISCU1/2, an essential component of iron-sulfur cluster assembly. Iron-sulfur clusters are crucial cofactors for TCA cycle aconitase, subunit D of succinate dehydrogenase, and ETC complexes I, II, and III. Thus, miR-210 upregulation under hypoxia inhibits the TCA cycle and OXPHOS (White et al., 2015). Hypoxia activates AMP-activated protein kinase (AMPK) by inhibiting mitochondrial OXPHOS, which in turn inhibits the PASMCs K_v_1.5 channel, inducing a hyperproliferative and anti-apoptotic phenotype in PASMCs (Moral-Sanz et al., 2016). AMPK can also downregulate PGC1-α expression in PAECs, resulting in decreased ATP production and increased ROS levels, leading to inhibition of mitochondrial oxidative metabolism and PAECs dysfunction (Ye et al., 2016). Abnormalities in mitochondrial oxygen metabolism, including diminished TCA cycling and inhibition of OXPHOS, characterize HPH. Although most current studies have focused on aerobic glycolysis in HPH, there are relatively few studies on mitochondrial oxygen metabolism. Further in-depth investigation is warranted to determine whether mitochondrial oxygen metabolism could be a potential therapeutic target and biomarker for HPH.

#### 3.3.5 MtROS in the development of HPH

A few of studies indicate that hypoxia has been shown to increase mitochondrial ROS (mtROS) production, while in others it leads to a decrease. This complexity arises from the different mechanisms influenced by hypoxia, such as increasing the lifetime of the semiquinone radical at the complex III site or increasing protein kinase C-β. Additionally, the relationship between mtROS and HIFs forms a negative feedback loop, which helps maintain mtROS homeostasis under hypoxia. Under normal conditions, a small fraction of electrons in the ETC are not transferred properly, producing ROS. MtROS, produced by complexes I, II, and III, are important for various cellular functions at physiological levels. However, excessive mtROS can cause cellular damage and contribute to pathological processes (Indo et al., 2017). The production and elimination of mtROS are regulated by enzymatic and non-enzymatic components, including superoxide dismutase (SOD), glutathione peroxidase (GPX), peroxidase (PRXS), catalase (CAT), and antioxidants such as tocopherols, ascorbic acid, reduced coenzyme Q10 and glutathione, but there is some debate about whether it increases or decreases production in hypoxia. Some studies have found decreased mtROS production during hypoxia (Michelakis et al., 2002a), while others have observed increased mtROS production in certain cell types (Bhansali et al., 2021; Siques et al., 2018; Korde et al., 2011). The change in mtROS production depends on factors such as the presence or absence of input block during hypoxia and the subunits of the ETC complex (Dunham-Snary et al., 2016). The increase in mtROS production during hypoxia may be due to the prolonged lifetime of the semiquinone radical at complex III’s external ubiquinone-binding site (Qo) (Waypa et al., 2013; Guzy et al., 2005). The RISP is also key for mtROS production in complex III under hypoxic conditions (Guzy et al., 2005; Korde et al., 2011), and complex I may also generate mtROS due to the inclusion of flavin mononucleotide (FMN)-linked sites and iron-sulfur clusters (Hirst et al., 2008; Fernández-Agüera et al., 2015). Furthermore, hypoxia increases mtROS production by upregulating protein kinase C-β (PKC-β) (Sheak et al., 2020). The release of mtROS into the cytoplasm enhances the activity of protein kinase C-ε and NADPH oxidase, which can contribute to a further increase in cytoplasmic ROS through ROS-induced ROS production (Rathore et al., 2006).

**FIGURE 4:**
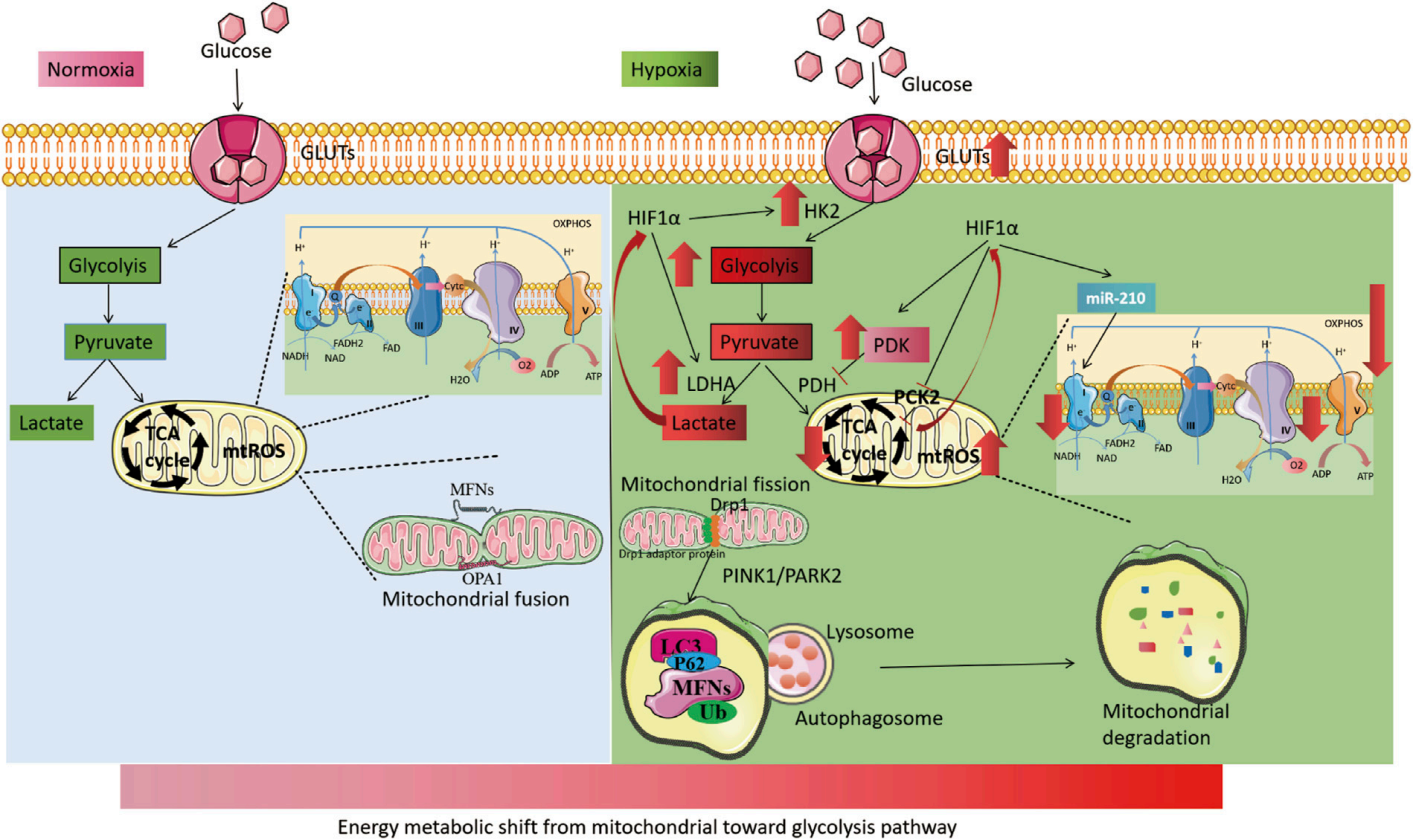
Mitochondrial changes under hypoxia. The left part illustrates the process of cellular energy metabolism under normoxia. Glucose enters the cell through glucose transporters (GLUTs) and undergoes glycolysis in the cytoplasm to produce pyruvate. Pyruvate then enters the mitochondria to participate in the TCA cycle and oxidative phosphorylation. Mitochondria maintain dynamic balance through continuous fission and fusion. The right part, shows the process of cellular energy metabolism under hypoxia. HIFs are activated, leading to an increase in the activity of GLUTs. Cellular energy metabolism is switched from mitochondria to glycolysis, with an increase in the activity of key glycolytic enzymes, including HK2 and LDHA. The activity of mitochondrial electron transport chain complex I and IV is reduced, and the TCA cycle is weakened (with increased PDK activity). Hypoxia also leads to increased mitochondrial fission and induces increased mitochondrial autophagy via the PINK1/PARK2 pathway.

Interestingly, mitochondria-dependent ROS signaling is involved in the process of hypoxia-induced transcriptional activation and plays a crucial role in the cellular hypoxic response. Hypoxia-induced mtROS generation may stabilize HIFs to regulate cellular hypoxic adaptation by inhibiting PHD2 activity through interaction with Fe^2+^ (Yang et al., 2020; Chandel et al., 2000). Additionally, exogenous peroxides (hydrogen peroxide or tert-butyl hydroperoxide) can inhibit FIH activity and regulate HIFs expression (Masson et al., 2012). Conversely, HIFs, as hypoxia-adaptive transcription factors, also affect mtROS production. HIFs reduce OXPHOS activity under hypoxia and decrease mtROS production. As previously described, HIFs reduce mtROS production by activating PDK1 activity to reduce pyruvate entry into the TCA cycle, upregulating NDUFA4L2 expression to reduce complex I activity, and enhancing miR-210 expression to inhibit ISCU1/2 activity (Tello et al., 2011; Zheng et al., 2022). These mechanisms form a negative feedback loop that regulates mtROS and HIFs, thereby maintaining mtROS homeostasis under hypoxia (Figure 4).

Hypoxia leads to changes in mtROS production, which in turn mediates hypoxic pulmonary vascular remodeling and PH by influencing the activity of PHD and regulating activation of HIFs. However, the role of altered mtROS levels in HPV and pulmonary vascular remodeling is a matter of debate, as mtROS are involved in hypoxic pulmonary vascular wall cell signaling through post-translational modifications of ion channels, protein kinases, and other signaling molecules. Acute hypoxia increases mtROS levels in PASMCs. MtROS inhibits K_v_1.5 ion channels on PASMCs through HIF1α activation, which causes membrane depolarization, opening of L-type calcium channels, increased calcium inward flow, and elevated cytoplasmic calcium ion concentration. These changes lead to PASMCs contraction (Waypa et al., 2013; Bonnet and Boucherat, 2018; Pak et al., 2018). The antioxidant Mitoquinone (MitoQ), which targets mitochondria, significantly inhibits HPV and acute hypoxia-induced elevated superoxide concentrations. In contrast, chronic hypoxia decreases mtROS levels in PASMCs but increases levels in the right ventricle. MitoQ does not affect chronic hypoxia-induced PH but attenuates chronic hypoxia-induced right ventricular remodeling, supporting the role of altered mtROS in chronic hypoxic PASMCs (Pak et al., 2018). Metabolic reprogramming in PASMCs during chronic hypoxia may account for reduced mtROS levels compared to acute hypoxia (Bonnet and Boucherat, 2018). Interestingly, hypoxia induces increased mtROS production in PASMCs in neonatal rats, but not in adult rats, suggesting that mtROS may be developmentally regulated (Sheak et al., 2020). Changes in mtROS production depend on the cell type. Chronic hypoxia increases mtROS levels in PAECs, promoting calcium inward flow via transient receptor potential 4 (TRV4) and activating the p38/mitogen-activated protein kinase (MAPK) pathway, ultimately increasing PAECs proliferation and migration. Both global ROS scavengers (TEMPOL) and mitochondria-specific antioxidants (MitoQ, CAT) can reduce mtROS levels, inhibit PAECs proliferation, and attenuate hypoxia-induced PH (Suresh et al., 2018; Adesina et al., 2015; Li et al., 2016). MtROS levels do not increase, or may even decrease, in pulmonary artery fibroblasts during acute and chronic hypoxia (Plecitá-Hlavatá et al., 2016; Waypa et al., 2013), but mtROS production in fibroblasts increases in PH due to downregulation of the auxiliary protein subunit of complex I, NDUFS4 (Plecitá-Hlavatá et al., 2016). This suggests that alterations in mitochondrial metabolism in fibroblasts may occur independently of hypoxia.

Together, there are conflicting results regarding the role of mtROS levels in HPH. This paradoxical effect may be influenced by various factors, including the experimental methods and equipment used to detect mtROS, the type of cells affected, the duration of hypoxia, and the involved intracellular signaling mechanisms.

**FIGURE 5:**
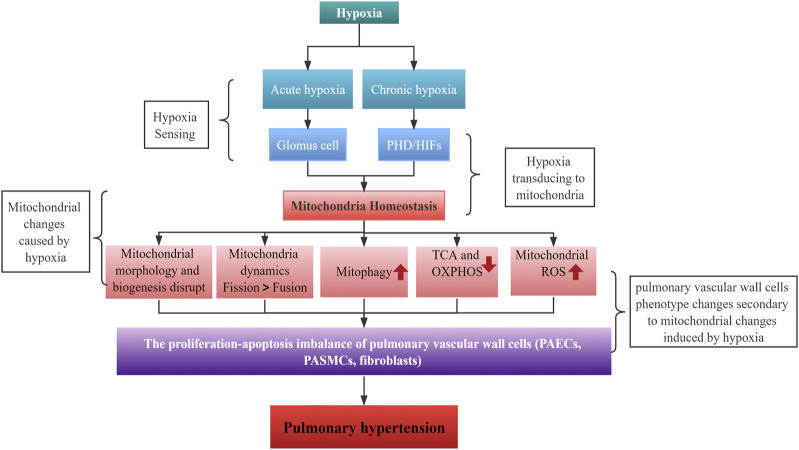
Schematic diagram of hypoxia causing PH by altering mitochondria.

Imbalances in mitochondrial homeostasis in HPH include disruption to mitochondrial dynamics (mainly increased fission due to fission/fusion imbalance), increased mitophagy, a metabolic shift toward aerobic glycolysis (Warburg effect), and abnormal mtROS production (Figure 5). Abnormalities originating from epigenetic regulation and/or post-transl translational modification of mitochondrial pathways are promising therapeutic targets for HPH in the future. Additional studies are necessary to gain a better understanding of the complex relationship between mitochondrial homeostasis and HPH. Investigating the specific roles and mechanisms of different mitochondrial proteins and signaling pathways in various cell types under hypoxic conditions will provide valuable insights into the pathogenesis of HPH.

Moreover, understanding the influence of factors such as age, disease stage, and comorbidities on mtROS production and mitochondrial homeostasis will help elucidate the role of mitochondria in the development and progression of this disease. Furthermore, the development of new therapeutic strategies targeting mitochondrial pathways in HPH is essential. This may involve using pharmacological agents or gene therapy to modulate mitochondrial dynamics, mitophagy, metabolism, and ROS production in affected cells. A combination of these approaches may be necessary to achieve optimal therapeutic effects and prevent or reverse the progression of HPH.

## 4 Targeting mitochondrial dysfunction for HPH and other PH

Mitochondrial dysfunction is present in many lung diseases. Therefore, targeting mitochondria has been applied to treat lung diseases such as COPD, asthma and in experimental animal models also (Agrawal and Mabalirajan, 2016). Mitochondrial dysfunction plays a crucial role in the development of HPH. Therefore, targeting this dysfunction may be essential in treating the condition. Various therapeutic agents have been used on both animal models and patients with PH, with mixed results (Table 2). The shift from OXPHOS to glycolysis contributes to the progression of HPH, making metabolic phenotype a promising target for treatment. Dichloroacetate (DCA), a PDK1 inhibitor, increases PDH activity and promotes acetyl coenzyme A entry into mitochondria, thus restoring cellular energy metabolism to OXPHOS. DCA has shown improvement in mPAP, right ventricular function, and mitochondrial respiration in animal models of PH, as well as functional capacity in patients with idiopathic pulmonary hypertension (IPAH) (Michelakis et al., 2017; McMurtry et al., 2004; Michelakis et al., 2002a; Michelakis et al., 2001; Cotroneo et al., 2015; Piao et al., 2013; Guo et al., 2020b). However, individual responses among IPAH patients vary, potentially due to functional variants in SIRT3 and UCP2 (Michelakis et al., 2017). The clinical efficacy of DCA in HPH patients remains unobserved. Cannabidiol, a compound from Cannabis sativa, can inhibit glycolysis and reverse abnormal metabolism by inhibiting the expression of GLUT1 and PDK1 to improve HPH (Lu et al., 2021). 3-Bromopyruvate, a selective HK2 inhibitor, reduces hypoxia-induced PH in animal models by inhibiting glycolysis and increasing mitochondrial respiration (Guo et al., 2020a; Chen et al., 2018b). Imatinib, a tyrosine kinase inhibitor, has been found to alleviate iron deficiency-induced PH by inhibiting GLUT1 activity, converting the metabolic phenotype (Cotroneo et al., 2015). Antioxidants are potential therapeutic agents for PH, as they help regulate mitochondrial redox homeostasis. Various antioxidants such as acetylcysteine, SOD, and EUK-134 have inhibited PH progression in animal models (Hoshikawa et al., 2001; Archer et al., 2010; Redout et al., 2010). However, MitoQ, which targets mtROS, does not affect chronic hypoxia-induced PH development but attenuates right ventricular remodeling (Pak et al., 2018). This supports the notion that increased mtROS may mediate HPV in acute hypoxic PASMCs but not chronic hypoxic pulmonary vascular remodeling. Increased mitochondrial fission and mitophagy are key factors in HPH development. FUNDC1 peptide inhibitors may reduce PH by inhibiting mitophagy under hypoxia (Liu et al., 2022). Mdivi-1, a Drp1 inhibitor, can improve exercise capacity, right ventricular function, and hemodynamics in experimental pulmonary hypertension by diminishing mitochondrial fission (Marsboom et al., 2012), but further data and application information are needed. Mitochondrial transplantation therapy is an emerging treatment strategy. One study found that the transfer of femoral artery smooth muscle cell mitochondria into PASMCs inhibited acute hypoxia-induced pulmonary vasoconstriction, and attenuated chronic hypoxia-induced pulmonary vascular remodeling (Zhu et al., 2016). Similarly, mitochondrial transplantation therapy has also been reported in the monocrotaline-induced PH rat model (Hsu et al., 2022). Oral exogenous mitochondria absorption into the bloodstream and entry into PASMCs using erythrocytes as carriers have also shown potential in attenuating hypoxia-induced PH (Xiao et al., 2022a). Despite these encouraging interventions, more studies are needed to determine if mitochondrial dysfunction-targeted interventions can improve PH survival and reverse pulmonary vascular remodeling. Other mitochondrial targets, such as mitochondrial biogenesis and mitophagy, have also been considered as therapeutic targets, but no relevant clinical or animal experiments have been reported.

**TABLE 2:** Clinical and animal experiments of mitochondrial intervention for pulmonary hypertension.

Mitochondrial pathway	Model	Drugs	Target	Therapeutic effect	References
Metabolism shift	Idiopathic pulmonary hypertension (IPAH) patients	DCA	PDK1	reduction in mean pulmonary artery pressure and pulmonary vascular resistance and improvement in functional capacity	Michelakis et al. (2017)
	monocrotaline-induced PH rat			decreased pulmonary vascular resistance, pulmonary artery medial thickness and right ventricular hypertrophy	McMurtry et al. (2004)
	Hypoxia-induced PH rat			prevented and reversed chronic HPH	Michelakis et al. (2002b), Michelakis et al. (2001), Piao et al. (2013)
	Iron-deficient induced PH rat			attenuated PH in iron deficient rat	Cotroneo et al. (2015)
	Monocrotaline-induced PH rat	3-Bromopyruvate	Hk2	improved pulmonary vascular remodeling and right ventricular function	Guo et al. (2020b)
	Hypoxia-induced PH rat			reversed the pulmonary vascular remodeling	Chen et al. (2018a)
	Iron-deficient induced PH rat	Imatinib	GLUT1	attenuated PH in iron deficient rat	Cotroneo et al. (2015)
	Hypoxia-induced PH mice	Cannabidiol	GLUT1/PDK1	recovering mitochondrial energy metabolism, normalizing the hypoxia-induced oxidant stress, reducing the lactate overaccumulation and abnormal glycolysis	Lu et al. (2021)
Mitochondrial dynamics	Hypoxia-induced PH rat	Mdivi-1	Drp-1	attenuated mPAP and pulmonary vascular remodeling	Chen et al. (2019)
	Hypoxia-induced PH rat			improved exercise capacity, right ventricular function and hemodynamics in experimental PH.	Marsboom et al. (2012)
Mitochondrial oxidative stress	Spontaneous PH fawn-hooded rat	SOD-mimetic (MnTBAP)	ROS	regressed PH	Archer et al. (2010)
	Monocrotaline-induced PH rat	EUK-134	ROS	attenuated pulmonary arterial hypertension-induced heart failure	Redout et al. (2010), Hoshikawa et al. (2001)
	Hypoxia-induced PH mice	MitoQ	MtROS	attenuated RV remodelling after chronic hypoxia	Pak et al. (2018)
Mitophagy	Hypoxia-induced PH mice	FUNDC1 peptide inhibitor	FUNDC1	reduced RVSP and increased PAAT in response to hypoxia	Liu et al. (2021)
Mitochondrial transplantation	Hypoxia-induced PH rat	Intravenous mitochondria	Mitochondria	Inhibited acute hypoxia-triggered pulmonary vasoconstriction, attenuated chronic hypoxia-induced pulmonary vascular remodeling	Zhu et al. (2016)
	Monocrotaline-induced PH rat			Potentiated medial remodeling and vasoreactivity of pulmonary artery, Improved right ventricular performance	Hsu et al. (2022)
	Hypoxia-induced PH rat	Orally-administrated mitochondria	Mitochondria	attenuated hypoxia- and monocrotaline-induced PH	Xiao et al. (2022b)

## 5 Future directions and challenges in targeting mitochondrial dysfunction for HPH

Targeting mitochondrial dysfunction in hypoxic pulmonary hypertension has shown promising results. However, there are still several challenges and areas for future research to optimize treatment outcomes: 1) Elucidating the precise mechanisms: Further investigation is needed to fully understand the mechanisms of mitochondrial dysfunction in hypoxic pulmonary hypertension. This includes the role of mtROS in different cell types, the role of mitochondrial dynamics, and the interplay between mitochondrial dysfunction and other signaling pathways. 2) Identifying novel therapeutic targets: As our understanding of mitochondrial dysfunction in hypoxic pulmonary hypertension expands, novel therapeutic targets are likely to emerge. These targets may include specific mitochondrial proteins, enzymes, or signaling pathways that contribute to the development and progression of the disease. 3) Developing targeted therapies: Current therapies targeting mitochondrial dysfunction have shown mixed results, highlighting the need for more targeted and specific interventions. The development of targeted therapies may require a personalized medicine approach, taking into account the individual patient’s genetic background and disease phenotype. 4) Improving drug delivery: Efficient drug delivery to the mitochondria remains a challenge. The development of novel drug delivery systems that specifically target mitochondria in pulmonary vascular cells may enhance therapeutic efficacy and reduce systemic side effects. 5) Combining therapies: Combination therapy, targeting multiple aspects of mitochondrial dysfunction and other pathophysiological pathways, may prove more effective in treating hypoxic pulmonary hypertension. Identifying synergistic drug combinations and optimal dosing regimens will be crucial in this regard. 6) Evaluating long-term safety and efficacy: As new therapies emerge, long-term studies will be necessary to assess their safety and efficacy in patients with hypoxic pulmonary hypertension. This includes evaluating the potential for adverse effects, such as mitochondrial toxicity, and monitoring for the development of drug resistance. 7) Translating findings from animal models to humans: Although animal models provide valuable insights into the pathogenesis of hypoxic pulmonary hypertension and potential therapeutic targets, translating these findings to human patients is challenging. Developing and validating appropriate preclinical models that accurately represent the human disease will be crucial for effective translation. By addressing these challenges and focusing on these future research directions, we may improve our understanding of the role of mitochondrial dysfunction in hypoxic pulmonary hypertension and develop more effective therapies for patients suffering from this devastating disease.

## 6 Conclusion and perspectives

Hypoxia is a natural process that activates the expression of multiple genes encoding proteins involved in cellular regulation, metabolism, survival, apoptosis, angiogenesis, and other functions through HIF activation. Mitochondria play a vital role in maintaining cellular energy metabolism by consuming oxygen and generating ROS. In response to hypoxia, mitochondria adapt by altering their morphological structure and dynamics, down-regulating the TCA cycle, modifying the activity of respiratory chain subunits, and adjusting mtROS production sources. There is substantial evidence indicating an interaction between mitochondrial signaling and HIF activity. Both TCA intermediates and mtROS are involved in regulating HIF activity, with HIFs being key mediators of hypoxia adaptation in the body and the core hub connecting hypoxia and mitochondrial metabolism. The lung is highly sensitive to oxygen content and the first organ to perceive changes in oxygen levels. Its adaptive response to hypoxia is involved in the occurrence and progression of lung diseases such as PH. Hypoxia regulates mitochondrial metabolism and mtROS, affecting target gene expression through the mitochondrial signaling pathway, leading to phenotypic changes in pulmonary artery wall cells and participating in the development of PH.

Therefore, targeting mitochondria may be a more reasonable approach to treating HPH. Mitochondrial metabolites, such as TCA cycle metabolic enzymes, can be used as markers to monitor disease progression and prognosis. However, further studies are needed to clarify the mechanism of hypoxia-induced mitochondrial changes involved in PH. Understanding these mechanisms will help identify more targets and may open up new therapeutic prospects for the treatment of HPH.
